# Associations between composite systemic inflammation indicators(CAR, CLR, SII, AISI, SIRI, and CALLY) and metabolic dysfunction-associated fatty liver disease (MAFLD): evidence from a two-stage study in China

**DOI:** 10.3389/fimmu.2025.1702567

**Published:** 2025-11-21

**Authors:** Jing Zhao, Quan Zhou, Yun Wang, Fangfei Xie, Jingyi Fan

**Affiliations:** Health Management Center, The Affiliated Suzhou Hospital of Nanjing Medical University, Suzhou Municipal Hospital, Gusu School, Nanjing Medical University, Suzhou, China

**Keywords:** metabolic dysfunction-associated fatty liver disease, immune-inflammatory indices, association, inflammation, risk

## Abstract

**Background:**

The redefinition from nonalcoholic fatty liver disease (NAFLD) to metabolic dysfunction-associated fatty liver disease (MAFLD) highlights metabolic dysfunction’s central role. While systemic inflammation drives MAFLD, large-scale evidence linking novel composite immune-inflammatory markers to MAFLD remains limited. This study evaluates six such markers and their association with MAFLD risk in a two-stage study in China.

**Methods:**

This two-stage study included a case-control analysis (7,894 MAFLD cases and matched controls) and a prospective cohort study (8,627 participants, median follow-up 2.37 years). Six composite immune inflammation-related markers (CAR, CLR, SII, AISI, SIRI, and CALLY) were derived from routine blood tests. Multivariable logistic and Cox regression models were used to assess associations with MAFLD risk, with adjustment for metabolic confounders. Restricted cubic splines (RCS) explored nonlinear relationships, and receiver operating characteristic (ROC) and decision curve analyses (DCA) evaluated predictive performance and clinical utility. The Cochran's Q test was used to evaluate the heterogeneity between groups to verify the influence of covariates.

**Results:**

All indicators show a significant nonlinear threshold effect relationship with the risk of MAFLD. CAR, CLR, SII, AISI, and SIRI exhibit a rapid increase in risk initially, followed by a plateau, while CALLY shows the opposite trend. Subgroup analysis indicates that SIRI is more strongly associated with MAFLD in women. Models combining these indicators with metabolic factors demonstrate superior predictive performance (AUC > 0.8) and clinical net benefit.

**Conclusion:**

Systemic immune-inflammatory indices exhibit nonlinear associations with MAFLD risk, independent of traditional metabolic factors. This reinforces MAFLD as a systemic inflammatory disease and highlights the potential of anti-inflammatory strategies, especially during the early, reversible stage before the risk plateaus.

## Introduction

Nonalcoholic fatty liver disease (NAFLD) affects 25-45% of the global population, with significant geographical variation (1). In Asia, rapid urbanization and demographic expansion have contributed to a NAFLD prevalence of 28–35%, posing a major public health challenge (2). In China, the prevalence rose from 17% in 2003 to 29.2% in 2018 and is projected to increase further, even if rates of obesity and diabetes remain stable (3, 4). With the rising burden of metabolic disorders and advancements in liver imaging, metabolic dysfunction-associated fatty liver disease (MAFLD) has replaced NAFLD as the preferred term for screening, diagnosis, and management of fatty liver disease (5, 6). Although studies have shown that more than 95% of patients with NAFLD meet the diagnostic criteria for MAFLD and that epidemiological data for NAFLD can be applied to MAFLD, they are not completely interchangeable (7, 8). The definition of MAFLD includes metabolic dysfunction and other causes of liver disease, covering a wider range of liver pathologies, which is the most significant difference from NAFLD.

The liver’s central role in lipid metabolism makes it particularly vulnerable to metabolic disturbances. Excessive lipid accumulation initiates a cascade of hepatic injury, triggering inflammatory responses that may progress to fibrosis and cirrhosis (6, 9). This pathogenic process is driven by oxidative stress and chronic inflammation, which facilitate the transition from simple fatty liver to steatohepatitis and advanced liver disease (10). The immune system, particularly through dysregulation of innate and adaptive immunity, plays a crucial role in mediating these inflammatory processes that underlie various liver pathologies (11, 12). Therefore, clarifying the relationship between inflammation and MAFLD is necessary for the prevention and treatment of MAFLD. Although previous studies have demonstrated associations between various immune-inflammatory markers and NAFLD (13, 14), the relationship between these indicators and MAFLD remains underexplored, particularly in case-control and cohort based studies since the adoption of the MAFLD diagnostic criteria.

This study aims to analyze and compare the relationship between MAFLD and six common composite immune inflammation-related indicators through case-control and cohort study designs in Chinese population.

## Materials and methods

### Study population

The participants of the first stage were recruited from January 2020 to November 2024 based on the Periodic Health Examination project at Suzhou Healthcare Center in southeastern China. A total of 30,190 participants aged over 18 years old received face to face interviews and complete physical examinations. We excluded the participants (1) with cancer and viral hepatitis at baseline (N = 184); (2) missing physical or laboratory examination (N = 1361); and (3) missing abdominal ultrasound (N = 1639). In the current study, 8,145 participants who had MAFLD were selected as cases. Controls were matched to the cases at a 1:1 ratio based on age and gender using the “MatchIt” package in R. The caliper was set to 0.2, meaning matches with a propensity score standard deviation exceeding 20% were discarded to ensure matching accuracy. Finally, we included 7,894 pairs of cases and controls in the analysis.

**Figure 1:**
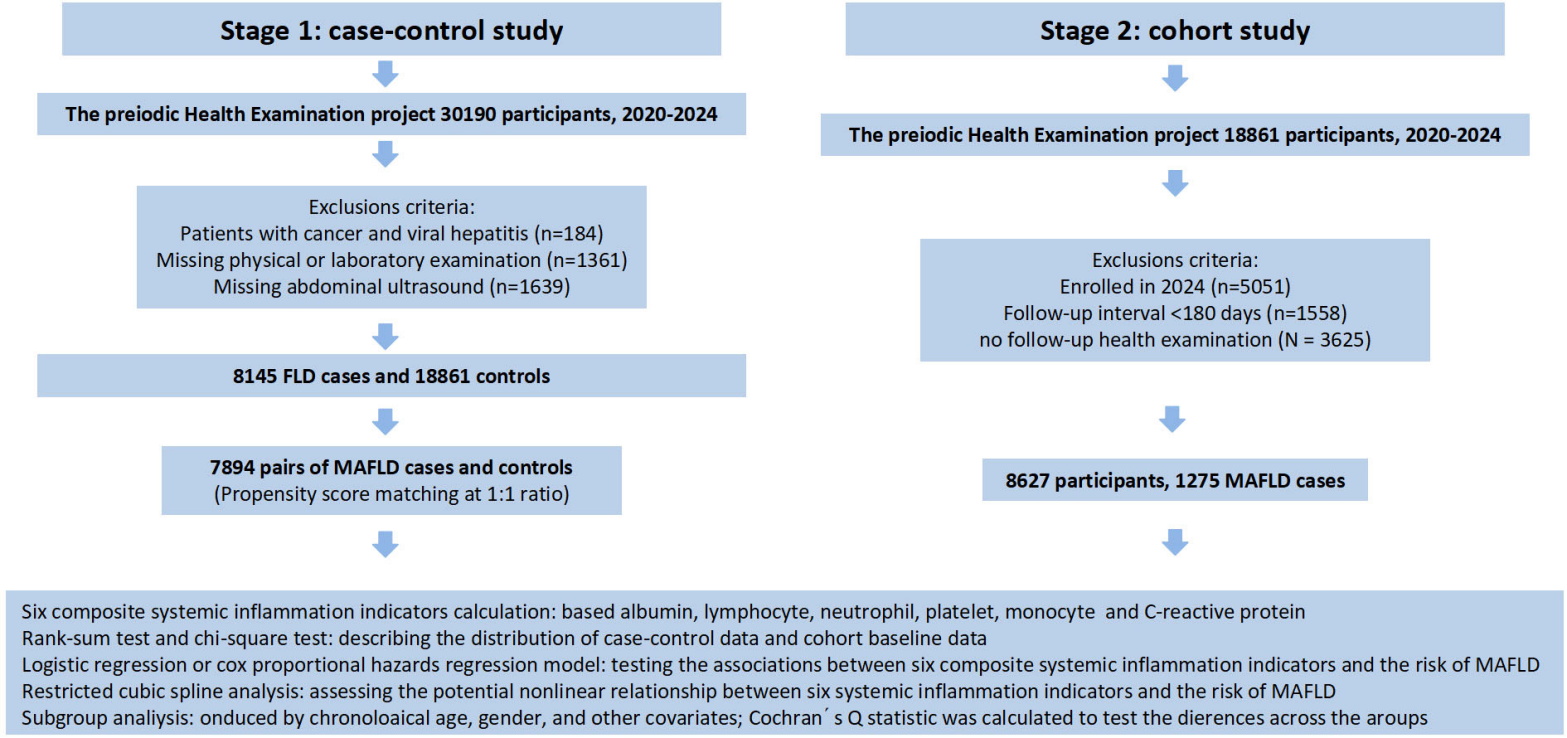
Flow chart of the study.

In the second stage of our study, we adopted a prospective cohort study design for the 18,861 participants without MAFLD in the first stage. Follow-up information was obtained from the participants’ periodic health examination. We excluded the following participants: (1) enrolled in 2024 as they had not yet reached the routine follow-up time (N = 5051); (2) follow-up interval <180 days (N = 1558); (3) no follow-up health examination (N = 3625). Ultimately, 8,627 participants were included in the subsequent analysis. The detailed screening process is illustrated in **Figure 1**.

This study was approved by the Ethics Committee of the Affiliated Suzhou Hospital of Nanjing Medical University (Ethical Approval Number: K-2025-061-K01), all participants received and signed written informed consent.

### Data collection

Physical examination data was obtained from hospital records at the time of enrollment. Information on smoking, alcohol consumption, tea drinking, and physical exercise was collected through face-to-face interviews. The questionnaire also covered medical history and medication use. Waist circumference (WC) was measured by an experienced surgeon approximately 2 cm above the participant’s umbilicus. Height and weight were automatically measured using the Omron ultrasonic weight scale HNH-318. Body mass index (BMI) was calculated as weight divided by the square of height (kg/m^2^).Blood samples were collected after a requested overnight fasting at least 8 hours. Complete blood count was measured using the Sysmex XN-20 automated modular hematology analyzer. Biochemical indicators such as liver functions were tested using the Beckman Coulter AU5800 automated biochemistry analyzer. Immune indicators such as C-reactive protein (CPR) were tested using the Immage 800 protein chemistry analyzer.

### Composite systemic inflammation indicators calculation

Composite systemic inflammation indicators are calculated by a series of immune and inflammation-related indicators, including albumin, lymphocyte, neutrophil, platelet, monocyte and CRP. Equation to calculate the six composite systemic inflammation indicators are shown below:


$$\text{CAR }(\text{CRP}-\text{to}-\text{albumin Ratio})=\frac{\text{CRP}}{\text{Albumin}}$$

(15)


$$\text{CLR}\;(\text{CRP}-\text{to}-\text{lymphocyte}\;\text{Ratio})=\frac{\text{CRP}}{\text{Lymphocyte}\;\text{count}}$$

(16)


$$\text{SII}\;(\text{SystemicImmune}-\text{inflammation}\;\text{Index})=\frac{\;\text{Neutrophil}\;\text{count}\times \;\text{Platelet}\;\text{count}\;}{\text{Lymphocyte}\;\text{count}}$$

(17)


$$\text{AISI}\;(\text{Aggregate}\;\text{Index}\;\text{of}\;\text{Systemic}\;\text{Inflammation})=\frac{\;\text{Neutrophil}\;\text{count}\;\times \;\text{Monocyte}\;\text{count}}{\text{Lymphocyte}\;\text{count}}$$

(18)


$$\text{SIRI }(\text{Systemic Inflammation Response Index})=\frac{\text{Neutrophil}\;\text{count}\;\times \;\text{Platelet}\;\text{count}\;\times \;\text{Monocyte}\;\text{count}}{\text{Lymphocyte}\;\text{count}}$$

(19)


$$\text{CALLY }(\text{CRP}-\text{Albumin}-\text{Lymphocyte Score})=\frac{\;\text{Albumin}\;\times \;\text{Lymphocyte}\;\text{count}}{\text{CRP}\;\times \;10}$$

(20)

### Metabolic dysfunction-associated fatty liver disease

The diagnosis of MAFLD was established with reference to the Guidelines for the Prevention and Treatment of MAFLD (2024 Edition) (5). First, the presence of hepatic steatosis is confirmed through imaging, and the diagnostic criteria for ultrasound are based on the guidelines issued by the Chinese Society of Hepatology in 2018 (21). Abdominal ultrasound was performed by trained and qualified clinical sonographer with GE HealthCare LOGIQ Premier-R2 and Philips EPIQ 7 color Doppler ultrasound systems. Next, fatty liver caused by genetic factors, medications, or alcohol consumption (ethanol intake ≥210g/week for men, ≥140g/week for women) is excluded. Finally, at least one of the following metabolic cardiovascular-related factors must be present:

1. BMI ≥ 24.0 kg/m², or waist circumference ≥ 90 cm (male) and 85 cm (female), or excessive body fat content and percentage.
2. Fasting Plasma Glucose (FPG) ≥ 6.1 mmol/L, or 2-hour post-load blood glucose ≥ 7.8 mmol/L, or a history of type 2 diabetes.
3. Fasting serum triglycerides ≥ 1.70 mmol/L, or currently receiving lipid-lowering medication; serum high-density lipoprotein ≤ 1.0 mmol/L (male) and 1.3 mmol/L (female), or currently receiving lipid-lowering medication.
4. Blood pressure ≥ 130/85 mmHg, or currently receiving antihypertensive medication.

This study also used fatty liver index (FLI) to quantitatively assess hepatic steatosis, and the formula is $\text{FLI}=\frac{{\text{e}}^{\text{x}}}{1+{\text{e}}^{\text{x}}}*100$.


X= 0.953×ln(TG)+0.139×BMI+0.718×ln(Gamma−Glutamyl Transferase)+0.053×WC–15.745

### Statistical analysis

The basic characteristics of the study participants were summarized using quartile for continuous variables and number (percentage) for categorical variables. Differences were evaluated using Wilcoxon test for continuous variables and Chi-square test for categorical variables. Logistic regression model and Cox proportional hazards regression model were used to assess the relationships between MAFLD and composite systemic inflammation indicators, as well as to estimate odds ratios (ORs) or hazard ratios (HRs) and 95% confidence intervals (95% CIs). Five models were constructed for analyses. Model 0 was unadjusted for covariates. Model 1 was adjusted for gender, age. Model 2 was further adjusted for hypertension, diabetes, BMI, WC, FPG, triglycerides (TG), total cholesterol (TC), high density lipoprotein-cholesterol (HDL-C) and low-density lipoprotein-cholesterol (LDL-C). Model 3 was further adjusted for aspartate aminotransferase (AST) and alanine aminotransferase (ALT) based on model 2. Model 4 was further adjusted for smoking and physical exercise based on model 2. Additionally, based on the quartiles of composite systemic inflammation indicators, participants were divided into four groups. Using the lowest quartile of each composite systemic inflammation indicator as the reference, the ORs and HRs of upper quartiles of each composite systemic inflammation indicator on MAFLD were calculated. Linear regression analysis of six composite systemic inflammation indicators on FLI index was conducted in case-control and cohort study. The receiver operating characteristic (ROC) was conducted to evaluate the discrimination performance of the six composite systemic inflammation indicators. The Youden index was calculated in order to find the optimal cut-off point and its corresponding sensitivity, specificity, positive predictive value(PPV) and negative predictive value(NPV).

Restricted cubic splines (RCS) were used to explore the shape of association between each composite systemic inflammation indicator and MAFLD with four knots placed at the 5th, 35th, 65th, and 95th percentiles. And the calibration curves and decision curve analysis (DCA) were conducted to evaluate the calibration and clinical usefulness of these models. The Cochran's Q test was used to evaluate the heterogeneity between groups to verify the influence of covariates on the association between MAFLD and the composite systemic inflammation indicator. The groups included gender, age (median), BMI (<28 kg/m²) (22), WC (<90 cm for men/<85 cm for women) (22), hypertension, diabetes, dyslipidemia and liver function enzymology (AST <40 U/L for men/<35 U/L for women; ALT: Male <50 U/L/female <40 U/L; Subgroup analysis was conducted for GGT<60 U/L. Hypertension is defined as meeting any one of the following conditions: (1) A doctor has diagnosed hypertension; (2) Currently taking antihypertensive drugs; (3) SBP≥140 mmHg or DBP≥90 mmHg. Diabetes is defined as meeting any of the following conditions: (1) A doctor has diagnosed diabetes; (2) Currently taking hypoglycemic drugs (oral or insulin); (3) Fasting blood glucose ≥7.0 mmol/L or HbA1c≥6.5%. Dyslipidemia is defined as meeting any one of the following conditions: (1) A doctor confirms dyslipidemia; (2) Currently taking lipid-lowering drugs; (3) TG≥2.26 mmol/L, or TC≥6.22 mmol/L, or LDL-C≥4.14 mmol/L, or HDL-C<1.04 mmol/L.

All the analyses were performed with R (version 4.4.3). Statistical significance was defined as two-sided *P* < 0.05.

## Results

### Baseline characteristics of participants

A total of 15,788 subjects were included in case-control study (**Table 1**), consisting of 11,954 (75.72%) males and 3,834(24.28%) females. Initial analysis revealed significant intergroup difference in CAR, CLR, SII, AISI, SIRI and CALLY between cases and controls (all *P* < 0.01). Compared with controls, MAFLD cases exhibited significantly elevated levels of CAR, CLR, SII, AISI and SIRI, along with reduced CALLY levels. Besides, MAFLD groups were higher in WC, BMI, AST, ALT, TC, TG, LDL-C, ablumin, CRP, neutrophil count, lymphocyte count, platelet count and monocyte count, while in HDL-C the opposite was true.

**Table 1:** Baseline characteristics of study population in the two-stage study.

Variables	Case-control analysis				Cohort analysis			
	Overall	Cases	Controls	*P* value	Overall	Incident	No incident	*P* value
N, n	15788 (100%)	7894 (50%)	7894 (50%)		8627 (100%)	1275 (14.78%)	7352 (85.22%)	
CAR	0.31 (0.18, 0.57)	0.40 (0.24, 0.72)	0.24 (0.15, 0.43)	**<0.01**	0.20 (0.13, 0.34)	0.27 (0.17, 0.46)	0.19 (0.12, 0.32)	**<0.01**
CLR	0.64 (0.37, 1.15)	0.79 (0.46, 1.40)	0.51 (0.31, 0.92)	**<0.01**	0.42 (0.27, 0.72)	0.55 (0.35, 0.92)	0.40 (0.26, 0.68)	**<0.01**
SII	358.27 (270.02, 477.25)	374.83 (281.56, 493.11)	344.15 (258.49, 460.75)	**<0.01**	357.35 (267.38, 471.91)	377.74 (286.30, 491.60)	354.05 (263.78, 467.47)	**<0.01**
AISI	0.70 (0.51, 0.97)	0.74 (0.54, 1.02)	0.66 (0.47, 0.92)	**<0.01**	0.64 (0.46, 0.88)	0.72 (0.52, 0.96)	0.62 (0.45, 0.86)	**<0.01**
SIRI	162.85 (110.89, 241.64)	178.31 (122.77, 261.76)	147.77 (101.75, 220.20)	**<0.01**	147.44 (101.71, 217.82)	166.42 (119.94, 237.99)	143.51 (99.04, 213.51)	**<0.01**
CALLY	6.92 (0.39, 12.25)	5.81 (3.23, 9.82)	8.77 (4.88, 14.36)	**<0.01**	10.51 (6.14, 16.50)	8.12 (4.79, 12.82)	11.05 (6.50, 17.19))	**<0.01**
Gender				1				**<0.01**
Male, n (%)	11954 (75.72%)	5977 (37.86%)	5977 (37.86%)		3714 (43.06%)	726 (8.42%)	2988 (34.64%)	
Female, n (%)	3834 (24.28%)	1917 (12.14%)	1917 (12.14%)		4913 (56.94%)	549 (6.36%)	4364 (50.58%)	
Age, years	44.90 (36.27, 54.69)	45.01 (36.28, 54.70)	44.84 (36.25, 54.69)	0.83	39.79 (32.05, 50.70)	43.20 (34.42, 53.72)	39.29 (31.79, 50.08)	**<0.01**
BMI, kg/m2	25.2 (23.2,27.5)	26.9 (25.1, 29.0)	23.7 (21.9, 25.4)	**<0.01**	22.7 (20.7, 24.6)	24.9 (23.3, 26.6)	22.3 (20.4, 24.1)	**<0.01**
WC, cm	87 (81, 93)	91 (87, 97)	83 (77, 89)	**<0.01**	79 (72, 85)	85 (81, 91)	77 (71, 83)	**<0.01**
AST, U/L	22.7 (19.0, 28.0)	24.9 (20.1, 31.3)	21.0 (18.0, 25.0)	**<0.01**	20.0 (17.0, 23.8)	20.8 (17.8, 25.0)	19.6 (16.8, 23.4)	**<0.01**
ALT, U/L	23.6 (16.4, 36.0)	30.0 (21.0, 46.8)	18.9 (14.0, 26.0)	**<0.01**	15.7 (11.5, 22.1)	19.0 (14.4, 27.3)	14.9 (11.0, 21.0)	**<0.01**
TC, mmol/L	5.13 (4.51, 5.82)	5.28 (4.65, 5.96)	5.00 (4.41, 5.67)	**<0.01**	4.89 (4.33, 5.52)	5.04 (4.48, 5.70)	4.87 (4.31, 5.48)	**<0.01**
TG, mmol/L	1.56 (1.09, 2.29)	1.96 (1.42, 2.78)	1.24 (0.91, 1.73)	**<0.01**	1.06 (0.78, 1.49)	1.49 (1.10, 2.07)	1.00 (0.75, 1.38)	**<0.01**
HDL-C, mmol/L	1.22 (1.06, 1.42)	1.15 (1.01, 1.32)	1.31 (1.13, 1.52)	**<0.01**	1.38 (1.19, 1.61)	1.23 (1.08, 1.40)	1.41 (1.22, 1.64)	**<0.01**
LDL-C, mmol/L	3.04 (2.58, 3.53)	3.15 (2.69, 3.64)	2.92 (2.49, 3.40)	**<0.01**	2.87 (2.44, 3.35)	3.10 (2.67, 3.57)	2.84 (2.41, 3.31)	**<0.01**
Hypertension				**<0.01**				**<0.01**
Yes, n (%)	2521 (15.97%)	1478 (9.36%)	1043 (6.61%)		1047 (12.14%)	287 (3.33%)	760 (8.81%)	
No, n (%)	13267 (84.03%)	6416 (41.64%)	6851 (43.39%)		7580 (87.86%)	988 (11.45%)	6592 (76.41%)	
Diabetes				**<0.01**				**<0.05**
Yes, n (%)	395 (1.9%)	224 (1.3%)	171 (0.6%)		180 (2.09%)	38 (0.44%)	142 (1.65%)	
No, n (%)	15393 (98.1%)	7670 (98.1%)	7923 (49.4%)		8447 (97.91%)	1237 (14.34%)	7210 (83.57%)	
Smoking				**<0.01**				**<0.01**
Yes, n (%)	3905 (24.73%)	2138 (27.08%)	1767 (22.38%)		727 (8.43%)	208 (16.31%)	519 (7.06%)	
No, n (%)	11883 (75.27%)	5756 (72.92%)	6127 (77.62%)		7900 (91.57%)	1067 (83.69%)	6833 (92.94%)	
Drinking				**<0.01**				0.24
Yes, n (%)	3256 (20.62%)	1738 (22.02%)	1518 (19.23%)		780 (9.04%)	127 (9.96%)	653 (8.88%)	
No, n (%)	12532 (79.38%)	6156 (77.98%)	6376 (80.77%)		7847 (90.96%)	1148 (90.04%)	6699 (91.12%)	
Exercise				**<0.01**				0.92
Yes, n (%)	11026 (69.84%)	5174 (65.54%)	5852 (74.13%)		5785 (67.06%)	853 (66.90%)	4932 (67.08%)	
No, n (%)	4762 (30.16%)	2720 (34.46%)	2042 (25.87%)		2842 (32.94%)	422 (33.10%)	2420 (32.92%)	
Albumin, g/L	45.2 (43.5, 46.9)	45.3 (43.7, 47.1)	45.0 (43.5, 46.7)	**<0.01**	44.6 (43.0, 46.4)	44.7 (43.2, 46.4)	44.6 (43.0, 46.4)	0.11
CRP, mg/L	1.40 (0.82, 2.58)	1.82 (1.09, 3.25)	1.08 (0.67, 1.94)	**<0.01**	0.89 (0.59, 1.51)	1.20 (0.78, 2.05)	0.85 (0.56, 1.41)	**<0.01**
Neutrophil count, 10^9^ cells/L	3.45 (2.82, 4.23)	3.66 (3.02, 4.44)	3.24 (2.66, 3.97)	**<0.01**	3.23 (2.64, 3.95)	3.51 (2.91, 4.20)	3.19 (2.60, 3.89)	**<0.01**
Lymphocyte count, 10^9^ cells/L	2.23 (1.84, 2.69)	2.34 (1.95, 2.82)	2.12 (1.76, 2.54)	**<0.01**	2.12 (1.76, 2.55)	2.22 (1.85, 2.69)	2.10 (1.75, 2.52)	**<0.01**
Platelet count, 10^9^ cells/L	235 (200, 272)	242 (206, 280)	228 (194, 264)	**<0.01**	234 (202, 273)	242 (206, 276)	233 (201, 272)	**<0.01**
Monocyte count, 10^9^ cells/L	0.46 (0.37, 0.56)	0.48 (0.40, 0.58)	0.43 (0.35, 0.53)	**<0.01**	0.42 (0.34, 0.51)	0.45 (0.37, 0.54)	0.41 (0.33, 0.50)	**<0.01**

Numerical variables are presented as median (lower quartile, upper quartile), and categorical variables are presented as count (percentage). P < 0.05 is bolded, indicating statistical significance.

CAR, CRP-to-albumin ratio; CLR, CRP-to-lymphocyte ratio; SII, Systemic immune-inflammation index; AISI, aggregate index of systemic inflammation; SIRI, systemic inflammation response index; CALLY, Controlling nutritional status and lymphocyte index; BMI, body mass index; WC, waist circumference; AST, aspartate aminotransferase; ALT, alanine aminotransferase; TG, triglycerides; TC, total cholesterol; HDL-C, high density lipoprotein-cholesterol; LDL-C, low density lipoprotein-cholesterol; CRP, C-reactive protein.

During median 2.37 years follow-up (IQR: 1.11-3.80), 1,275 incident MAFLD cases were identified from 8,627 eligible participants in the cohort study (**Table 1**). Among them, CAR, CLR, SII, AISI, and SIRI were higher in the incident group than in the non-incident group, while CALLY was lower in the incident group than in the non-incident group. Participants with MAFLD incident were predominantly male and demonstrated adverse metabolic profiles, including elevated BMI, WC, AST, ALT, TC, TG, LDL-C, CRP, neutrophil count, lymphocyte count, platelet count and monocyte count, decreased HDL-C (all *P* < 0.01).Differential analysis found there no significant intergroup difference in CAR, CLR, SII, AISI, SIRI and CALLY between participants lost to follow-up and those who completed follow-up (all *P*>0.05) (**Supplementary Table 1**).

### Associations between MAFLD and composite systemic inflammation indicators (CAR, CLR, SII, AISI, SIRI and CALLY)

**Figure 2:**
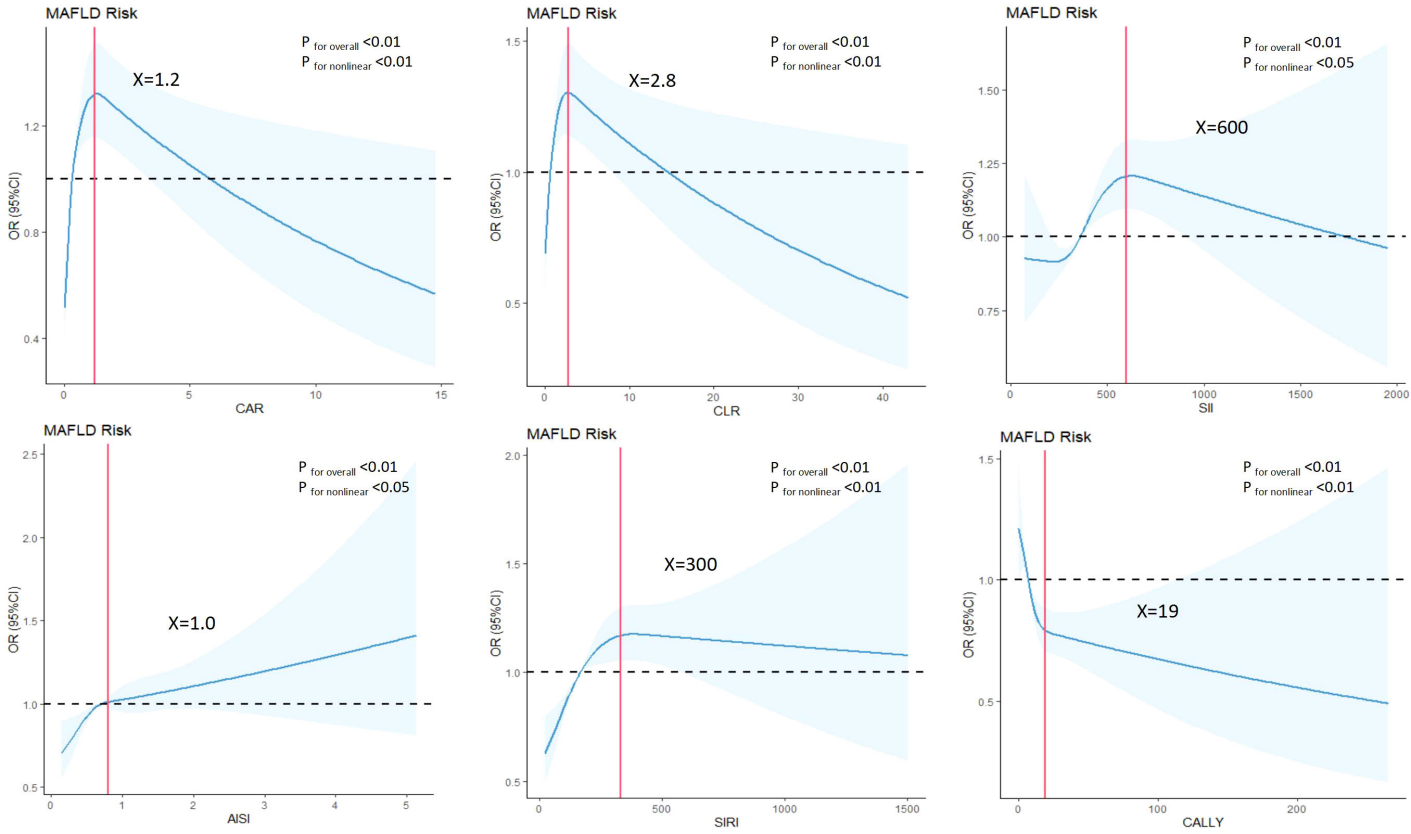
Associations between composite systemic inflammation indicators (CAR, CLR, SII, AISI, SIRI and CALLY) and MAFLD risk in case-control study. ORs (95%CI) were derived from restricted cubic spline regression, with knots placed at the 5th, 35th, 65th and 95th percentiles ofcomposite systemic inflammation indicators. Panels were adjusted for the same variables as model 2 in **Table 2**.

In the case-control study (**Table 2**), CAR, CLR, SII, AISI, SIRI and CALLY were significantly associated with increased risk of MAFLD per-sd increase in the unadjusted model. This association persisted with adjustment for gender, age. Further adjustment for additional covariates (history of hypertension and diabetes, BMI, WC, FPG and blood lipids) attenuated the effect size, but CAR, SII, AISI, SIRI and CALLY remained statistically significant. Moreover, we observed a strong dose-response relationship (*P* for trend < 0.01) across increasing quartiles of CAR, CLR, SII, AISI, SIRI and CALLY. Compared with the participants in the lowest quartile group, those in the top quartile group had higher risks of MAFLD with ORs, expect for CALLY. To enhance the robustness of our findings, non-linear relationships between composite systemic inflammation indicators and MAFLD risk were conducted using RCS model, adjusting additional confounders. Significant non-linear associations were identified for CAR, CLR, SII, AISI, SIRI and CALLY with MAFLD risk (*P* for non-linear<0.01) (**Figure 2**). While CAR, CLR, SII, AISI, and SIRI showed steep risk increases up to respective thresholds (1.2, 2.8, 600, 1.0, 300) followed by plateauing, CALLY displayed an inflection point at approximately 19, transitioning from a steep decline to a much weaker negative trend. There are also linear relationships between CAR, SII, AISI, SIRI with FLI after adjusting gender, age, hypertension and diabetes, BMI, WC, FPG and blood lipids.

**Table 2:** Associations between six composite systemic inflammation indicators (CAR, CLR, SII, AISI, SIRI and CALLY) and MAFLD risk in the case-control study.

Case-control	Cases/controls	Model 0	*P* value	Model 1	*P* value	Model 2	*P* value
CAR							
CAR per-sd increase	7894/7894	1.43 (1.36, 1.51)	**<0.01**	1.41 (1.34, 1.49)	**<0.01**	1.04 (1.00, 1.08)	**<0.01**
Q1	1137/2807	Reference	Reference	Reference	Reference	Reference	Reference
Q2	1842/2110	2.16 (1.96, 2.37)	**<0.01**	2.16 (1.97, 2.37)	**<0.01**	1.42 (1.26, 1.59)	**<0.01**
Q3	2230/1715	3.21 (2.92, 3.52)	**<0.01**	3.29 (2.93, 3.53)	**<0.01**	1.66 (1.49, 1.87)	**<0.01**
Q4	2685/1262	5.25 (4.77, 5.78)	**<0.01**	5.27 (4.79, 5.80)	**<0.01**	1.87 (1.66, 2.11)	**<0.01**
*P* for trend			**<0.01**		**<0.01**		**<0.01**
CLR							
CLR per-sd increase	7894/7894	1.09 (1.07, 1.12)	**<0.01**	1.09 (1.07, 1.11)	**<0.01**	1.01 (0.99, 1.03)	0.34
Q1	1317/2632	Reference	Reference	Reference	Reference	Reference	Reference
Q2	1817/2128	1.71 (1.56, 1.87)	**<0.01**	1.71 (1.56, 1.87)	**<0.01**	1.19 (1.06, 1.33)	**<0.01**
Q3	2180/1766	2.47 (2.25, 2.70)	**<0.01**	2.48 (2.26, 2.71)	**<0.01**	1.33 (1.18, 1.49)	**<0.01**
Q4	2580/1368	3.77 (3.43, 4.14)	**<0.01**	3.80 (3.46, 4.17)	**<0.01**	1.59 (1.42, 1.79)	**<0.01**
*P* for trend			**<0.01**		**<0.01**		**<0.01**
SII							
SII per-sd increase	7894/7894	1.001 (1.001, 1.001)	**<0.01**	1.001 (1.001, 1.001)	**<0.01**	1.000 (1.000, 1.001)	**<0.01**
Q1	1746/2201	Reference	Reference	Reference	Reference	Reference	Reference
Q2	1865/2082	1.13 (1.03, 1.23)	**<0.01**	1.13 (1.03, 1.23)	**<0.01**	0.97 (0.87, 1.09)	0.62
Q3	2111/1836	1.45 (1.32, 1.58)	**<0.01**	1.46 (1.33, 1.59)	**<0.01**	1.16 (1.04, 1.29)	**<0.01**
Q4	2172/1775	1.54 (1.41, 1.69)	**<0.01**	1.55 (1.42, 1.70)	**<0.01**	1.16 (1.03, 1.29)	**<0.05**
*P* for trend			**<0.01**		**<0.01**		**<0.01**
AISI							
AISI per-sd increase	7894/7894	1.48 (1.38, 1.59)	**<0.01**	1.46 (1,36, 1.57)	**<0.01**	1.16 (1.06, 1.26)	**<0.01**
Q1	1652/2374	Reference	Reference	Reference	Reference	Reference	Reference
Q2	1972/2053	1.39 (1.27, 1.52)	**<0.01**	1.40 (1.28, 1.52)	**<0.01**	1.12 (1.00, 1.25)	**<0.05**
Q3	2162/1862	1.68 (1.54, 1.84)	**<0.01**	1.69 (1.55, 1.86)	**<0.01**	1.20 (1.08, 1.35)	**<0.01**
Q4	2265/1761	1.89 (1.73, 2.07)	**<0.01**	1.91 (1.75, 2.09)	**<0.01**	1.23 (1.10, 1.38)	**<0.01**
*P* for trend			**<0.01**		**<0.01**		**<0.01**
SIRI							
SIRI per-sd r increase	7894/7894	1.002 (1.002, 1.002)	**<0.01**	1.002 (1.002, 1.002)	**<0.01**	1.001 (1.00, 1.001)	**<0.01**
Q1	1548/2399	Reference	Reference	Reference	Reference	Reference	Reference
Q2	1889/2058	1.42 (1.30, 1.56)	**<0.01**	1.43 (1.31, 1.56)	**<0.01**	1.16 (1.03, 1.29)	**<0.05**
Q3	2131/1816	1.81 (1.66, 1.99)	**<0.01**	1.83 (1.68, 2.01)	**<0.01**	1.29 (1.16, 1.45)	**<0.01**
Q4	2326/1621	2.22 (2.03, 2.43)	**<0.01**	2.25 (2.05, 2.46)	**<0.01**	1.42 (1.26, 1.69)	**<0.01**
*P* for trend			**<0.01**		**<0.01**		**<0.01**
CALLY							
CALLY per-sd increase	7894/7894	0.95 (0.95, 0.95)	**<0.01**	0.95 (0.95, 0.95)	**<0.01**	0.990 (0.986, 0.995)	**<0.01**
Q1	2559/1387	Reference	Reference	Reference	Reference	Reference	Reference
Q2	2172/1774	0.66 (0.61, 0.73)	**<0.01**	0.67 (0.60, 0.72)	**<0.01**	0.86 (0.77, 0.97)	**<0.05**
Q3	1834/2112	0.47 (0.43, 0.52)	**<0.01**	0.47 (0.43, 0.52)	**<0.01**	0.78 (0.70, 0.88)	**<0.01**
Q4	1328/2618	0.27 (0.25, 0.30)	**<0.01**	0.27 (0.25, 0.28)	**<0.01**	0.65 (0.58, 0.74)	**<0.01**
*P* for trend			**<0.01**		**<0.01**		**<0.01**

Data are presented as count, OR (95%CI). P < 0.05 is bolded, indicating statistical significance.

Model 1 was adjusted for gender and age; Model 2 was further adjusted for hypertension, diabetes, BMI, WC, TC, TG, HDL-C, LDL-C, FPG.

CAR, CRP-to-albumin ratio; CLR, CRP-to-lymphocyte ratio; SII, Systemic immune-inflammation index; AISI, aggregate index of systemic inflammation; SIRI, systemic inflammation response index; CALLY, Controlling nutritional status and lymphocyte index; BMI, body mass index; WC, waist circumference; TG, triglycerides; TC, total cholesterol; HDL-C, high density lipoprotein-cholesterol; LDL-C, low density lipoprotein-cholesterol; CRP, C-reactive protein.

**Figure 3:**
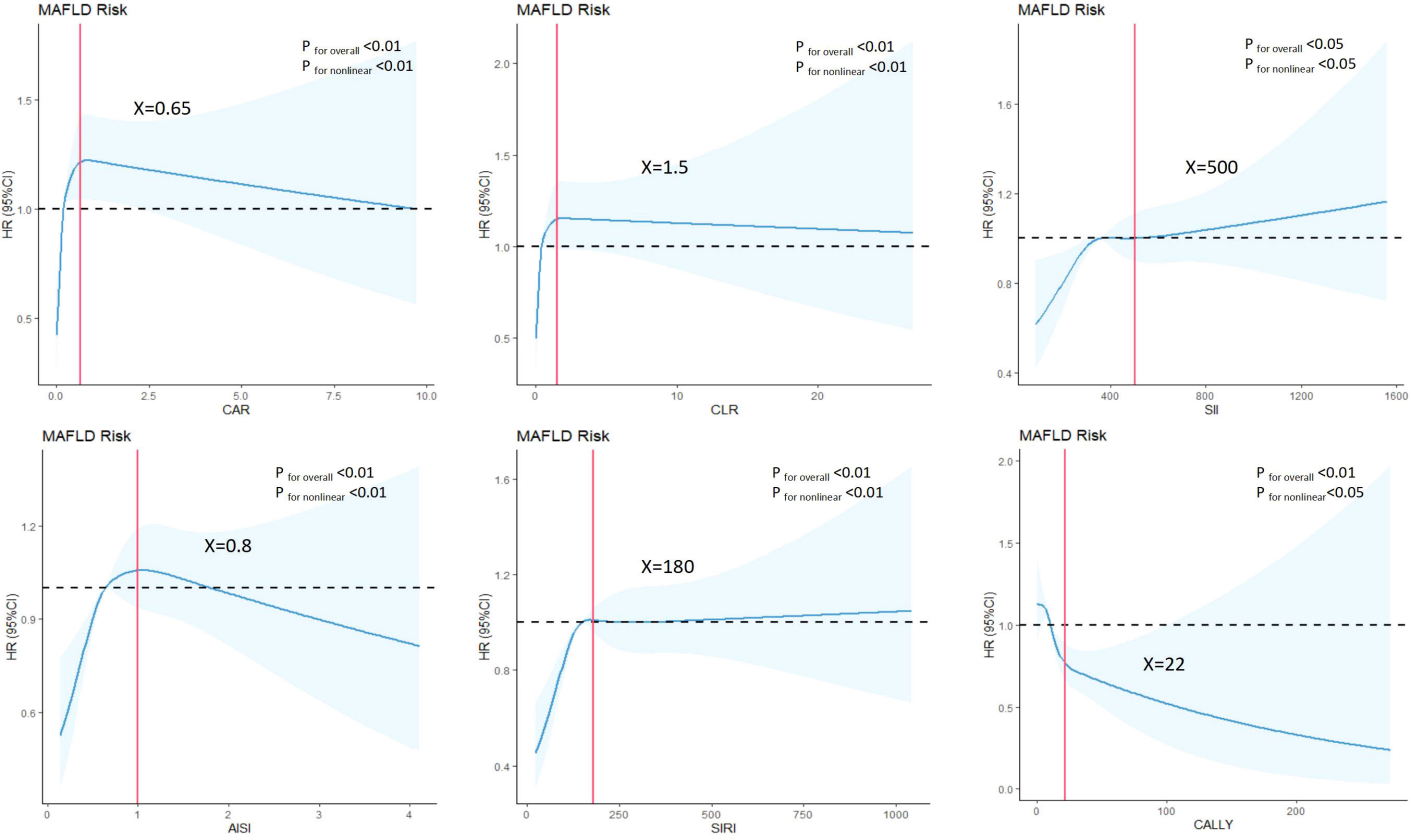
Associations between composite systemic inflammation indicators (CAR, CLR, SII, AISI, SIRI and CALLY and MAFLD risk in cohort study. HRs (95%CI) were derived from restricted cubic spline regression, with knots placed at the 5th, 35th, 65th and 95th percentiles of composite systemic inflammation indicators. Panels were adjusted for the same variables as model 2 in **Table 3**.

In the cohort study (**Table 3**), each sd increase in CAR, CLR, SII, AISI, SIRI and CALLY were significantly associated with elevated MAFLD risk in the crude model. After adjusting for gender, age, the association was attenuated. Further adjustment for hypertension, diabetes, BMI, WC, and blood lipids led to additional attenuation, only the association in SII, SIRI and CALLY still reached statistical significance. Furthermore, significant dose-response relationship was evident across ascending quartiles of CAR, CLR, SII, AISI, SIRI and CALLY (*P* for trend < 0.01). RCS models were further conducted to find whether nonlinear associations existed between composite systemic inflammation indicators and MAFLD risk. As shown in **Figure 3**, there were significant nonlinear relationships between CAR, CLR, SII, AISI and SIRI and MAFLD after adjusting additional confounders (*P* for nonlinear < 0.05).The risks associated with CAR, CLR, SII, AISI, and SIRI increased steeply until reaching approximate thresholds of 0.65, 1.5, 500, 0.8, and 180, respectively, beyond which the curves flattened. But the CALLY indicator exhibits an inflection point at approximately 22, transitioning from a steep decline to an attenuated negative trend. There are also linear relationships between CAR, AISI with FLI after adjusting gender, age, hypertension and diabetes, BMI, WC, FPG and blood lipids (**Table 4**).

**Table 3:** Associations between six composite systemic inflammation indicators (CAR, CLR, SII, AISI, SIRI and CALLY) and MAFLD risk in the cohort study.

Cohort	N/Cases	Person-years	Model 0	*P* value	Model 1	*P* value	Model 2	*P* value
CAR								
CAR per-sd increase	8627/1275	21352	1.06 (1.04, 1.09)	**<0.01**	1.05 (1.03, 1.08)	**<0.01**	1.02 (0.98, 1.06)	0.25
Q1	2157/138	5532	Reference	Reference	Reference	Reference	Reference	Reference
Q2	2166/278	5472	1.99 (1.63, 2.45)	**<0.01**	1.83 (1.49, 2.25)	**<0.01**	1.40 (1.14, 1.73)	**<0.01**
Q3	2147/371	5284	2.78 (2.28, 3.38)	**<0.01**	2.52 (2.07, 3.08)	**<0.01**	1.49 (1.22, 1.82)	**<0.01**
Q4	2157/488	5065	4.10 (3.39, 4.95)	**<0.01**	3.56 (2.94, 4.32)	**<0.01**	1.79 (1.47, 2.18)	**<0.01**
*P* for trend				**<0.01**		**<0.01**		**<0.01**
CLR								
CLR per-sd increase	8627/1275	21352	1.07 (1.04, 1.10)	**<0.01**	1.06 (1.03, 1.09)	**<0.05**	1.03 (0.99, 1.07)	0.19
Q1	170/2161	5520	Reference	Reference	Reference	Reference	Reference	Reference
Q2	284/2153	5458	1.65 (1.38, 2.02)	**<0.01**	1.55 (1.28, 1.88)	**<0.01**	1.21 (1.00, 1.47)	0.05
Q3	372/2156	5266	2.36 (1.97, 2.83)	**<0.01**	2.19 (1.83, 2.64)	**<0.01**	1.50 (1.24, 1.80)	**<0.01**
Q4	449/2157	5108	3.06 (2.57, 3.66)	**<0.01**	2.73 (2.27, 3.27)	**<0.01**	1.51 (1.26, 1.82)	**<0.01**
*P* for trend				**<0.01**		**<0.01**		**<0.01**
SII								
SII per-sd increase	8627/1275	21352	1.10 (1.05 1.15)	**<0.01**	1.08 (1.03, 1.13)	**<0.01**	1.06 (1.01, 1.12)	**<0.05**
Q1	245/2157	5252	Reference	Reference	Reference	Reference	Reference	Reference
Q2	324/2157	5304	1.39 (1.18, 1.64)	**<0.01**	1.34 (1.14, 1.58)	**<0.01**	1.24 (1.05, 1.46)	**<0.05**
Q3	337/2156	5292	1.55 (1.32, 1.83)	**<0.01**	1.45 (1.23, 1.71)	**<0.01**	1.39 (1.18, 1.64)	**<0.01**
Q4	369/2157	5504	1.54 (1.31, 1.81)	**<0.01**	1.41 (1.20, 1.66)	**<0.01**	1.27 (1.08, 1.49)	**<0.01**
*P* for trend				**<0.01**		**<0.01**		**<0.05**
AISI								
AISI per-sd increase	8627/1275	21352	1.13 (1.09, 1.17)	**<0.01**	1.08 (1.04, 1.12)	**<0.01**	1.04 (0.99, 1.09)	0.09
Q1	200/2157	5294	Reference	Reference	Reference	Reference	Reference	Reference
Q2	299/2157	5292	1.53 (1.28, 1.83)	**<0.01**	1.46 (1.22, 1.75)	**<0.01**	1.27 (1.06, 1.52)	**<0.05**
Q3	371/2156	5324	1.96 (1.65, 2.33)	**<0.01**	1.81 (1.52, 2.15)	**<0.01**	1.38 (1.16, 1.64)	**<0.01**
Q4	405/2157	5442	2.07 (1.74, 2.45)	**<0.01**	1.76 (1.48, 2.09)	**<0.01**	1.41 (1.18, 1.68)	**<0.01**
*P* for trend				**<0.01**		**<0.01**		**<0.01**
SIRI								
SIRI per-sd r increase	8627/1275	21352	1.12 (1.08, 1.16)	**<0.01**	1.10 (1.06, 1.14)	**<0.01**	1.06 (1.01, 1.11)	**<0.05**
Q1	203/2157	5290	Reference	Reference	Reference	Reference	Reference	Reference
Q2	305/2157	5304	1.60 (1.34, 1.91)	**<0.01**	1.56 (1.30, 1.86)	**<0.01**	1.38 (1.16, 1.66)	**<0.01**
Q3	369/2156	5366	1.90 (1.60, 2.26)	**<0.01**	1.83 (1.54, 2.17)	**<0.01**	1.41 (1.19, 1.68)	**<0.01**
Q4	598/2157	5392	2.00 (1.69, 2.36)	**<0.01**	1.88 (1.58, 2.23)	**<0.01**	1.49 (1.25, 1.76)	**<0.01**
*P* for trend				**<0.01**		**<0.01**		**<0.01**
CALLY								
CALLY per-sd increase	8627/1275	21352	0.36 (0.30, 0.43)	**<0.01**	0.41 (0.34, 0.49)	**<0.01**	0.73 (0.62, 0.85)	**<0.01**
Q1	451/2157	5134	Reference	Reference	Reference	Reference	Reference	Reference
Q2	370/2157	5264	0.76 (0.67, 0.88)	**<0.01**	0.80 (0.70, 0.92)	**<0.01**	0.94 (0.82, 1.08)	0.4
Q3	281/2156	5442	0.54 (0.47, 0.63)	**<0.01**	0.57 (0.49, 0.66)	**<0.01**	0.81 (0.69, 0.95)	**<0.01**
Q4	173/2157	5513	0.34 (0.28, 0.40)	**<0.01**	0.37 (0.31, 0.45)	**<0.01**	0.67 (0.55, 0.80)	**<0.01**
*P* for trend				**<0.01**		**<0.01**		**<0.01**

Data are presented as count, HR (95%CI).

Model 1 was adjusted for gender and age; Model 2 was further adjusted for hypertension, diabetes, BMI, WC, TC, TG, HDL-C, LDL-C, FPG.

CAR, CRP-to-albumin ratio; CLR, CRP-to-lymphocyte ratio; SII, Systemic immune-inflammation index; AISI, aggregate index of systemic inflammation; SIRI, systemic inflammation response index; CALLY, Controlling nutritional status and lymphocyte index; BMI, body mass index; WC, waist circumference; TG, triglycerides; TC, total cholesterol; HDL-C, high density lipoprotein-cholesterol; LDL-C, low density lipoprotein-cholesterol; CRP, C-reactive protein.

**Table 4:** Linear Regression Analysis of six composite systemic inflammation indicators (CAR, CLR, SII, AISI, SIRI and CALLY) on FLI index in case-control and cohort study.

Indicators	Case-control study						Cohort study					
	Rough model			Adjusted model			Rough model			Adjusted model		
	B	Beta	*P* value	B	Beta	*P* value	B	Beta	*P* value	B	Beta	*P* value
CAR	3.07	0.12	**<0.01**	0.19	0.10	**<0.05**	2.56	0.11	**<0.01**	0.31	0.01	**<0.05**
CLR	0.77	0.07	**<0.01**	0.03	0	0.52	0.78	0.08	**<0.01**	0.06	0.01	0.25
SII	0.01	0.05	**<0.01**	0	0.01	0.18	0	0.01	0.31	0	0	0.71
AISI	7.70	0.14	**<0.01**	0.93	0.02	**<0.01**	5.83	0.14	**<0.01**	0.45	0.01	**<0.05**
SIRI	0.03	0.15	**<0.01**	0	0.02	**<0.01**	0.02	0.11	**<0.01**	0	0.01	0.06
CALLY	-0.29	-0.17	**<0.01**	-0.03	-0.02	**<0.01**	-0.10	-0.13	**<0.01**	-0.01	-0.01	0.22

Adjusted model was adjusted for gender, age, hypertension, diabetes, BMI, WC, TC, TG, HDL-C, LDL-C.

FLI, fatty liver index; CAR, CRP-to-albumin ratio; CLR, CRP-to-lymphocyte ratio; SII, Systemic immune-inflammation index; AISI, aggregate index of systemic inflammation; SIRI, systemic inflammation response index; CALLY, Controlling nutritional status and lymphocyte index; BMI, body mass index; WC, waist circumference; TG, triglycerides; TC, total cholesterol; HDL-C, high density lipoprotein-cholesterol; LDL-C, low density lipoprotein-cholesterol.

In sensitivity analyses, even after adjusting for liver enzymes (ALT and AST) or smoking and physical activity based on Model 2, the observed results remained similar (**Supplementary Tables 2**, **3**).

### Predictive ability of composite systemic inflammation indicators (CAR, CLR, SII, AISI, SIRI, and CALLY) for MAFLD

Compared with rough model and adjusted model, comprehensive model of CAR, CLR, SII, AISI, SIRI, and CALLY showed higher AUCs over 0.8. The sensitivity of comprehensive model for predicting MAFLD of these indicators ranged from 78.2% to 82.2% and the specificity range from 65.8% to 70.9%. The comprehensive model of these indicators yielded positive predictive values (PPV) and negative predictive values (NPV) for MAFLD ranging from 29.4% to 31.5% and 94.7% to 95.5%, respectively (**Table 5**).

**Table 5:** Analysis of the diagnostic ability of six composite systemic inflammation indicators (CAR, CLR, SII, AISI, SIRI and CALLY) for MAFLD in cohort study.

Indicators	AUC (95%CI)	Optimal cut-off	Sensitivity (%)	Specificity (%)	PPV	NPV
Rough model						
CAR	0.64 (0.62, 0.66)	0.22	62.40	59.17	20.96	90.08
CLR	0.61 (0.60, 0.63)	0.41	66.90	50.20	18.90	89.70
SII	0.54 (0.53, 0.56)	355.15	56.80	50.30	16.50	87.00
AISI	0.58 (0.57, 0.60)	0.65	59.10	54.00	18.20	88.40
SIRI	0.58 (0.56, 0.60)	133.06	68.40	44.90	17.70	89.10
CALLY	0.61 (0.59, 0.63)	10.87	33.73	48.98	10.28	80.99
Adjusted model						
CAR	0.53 (0.51, 0.55)	-0.20	62.53	25.78	12.92	80.30
CLR	0.53 (0.51, 0.55)	-0.51	68.24	20.24	12.92	78.61
SII	0.52 (0.50, 0.53)	-57.95	59.84	43.82	15.59	86.29
AISI	0.52 (0.50, 0.53)	0.02	40.16	64.58	16.43	86.15
SIRI	0.52 (0.50, 0.54)	-30.79	53.41	50.34	15.72	86.17
CALLY	0.52 (0.51, 0.54)	-5.10	71.67	36.40	16.16	87.74
Comprehensive model						
CAR	0.81 (0.80, 0.82)	0.14	78.20	69.72	30.93	94.86
CLR	0.81 (0.80, 0.82)	0.14	77.10	70.93	31.51	94.70
SII	0.81 (0.80, 0.82)	0.12	82.20	65.82	29.43	95.52
AISI	0.81 (0.80, 0.82)	0.14	79.14	68.84	30.58	95.01
SIRI	0.81 (0.80, 0.82)	0.13	79.53	68.61	30.52	95.08
CALLY	0.81 (0.80, 0.82)	0.14	78.20	69.80	30.99	94.86

CAR, CRP-to-albumin ratio; CLR, CRP-to-lymphocyte ratio; SII, Systemic immune-inflammation index; AISI, aggregate index of systemic inflammation; SIRI, systemic inflammation response index; CALLY, Controlling nutritional status and lymphocyte index; AUC, Area under circle; PPV, Positive predictive value; NPV, Negative predictive value.

Adjusted model was conducted by residual adjustment method to adjusted for demographic factors (gender and age) and metabolism-related factors (hypertension, diabetes, BMI, WC, TC, TG, HDL-C, LDL-C). Comprehensive model was conducted by multivariable logistic regression, including demographic factors (gender and age) and metabolism-related factors (hypertension, diabetes, BMI, WC, TC, TG, HDL-C, LDL-C).

**Figure 4:**
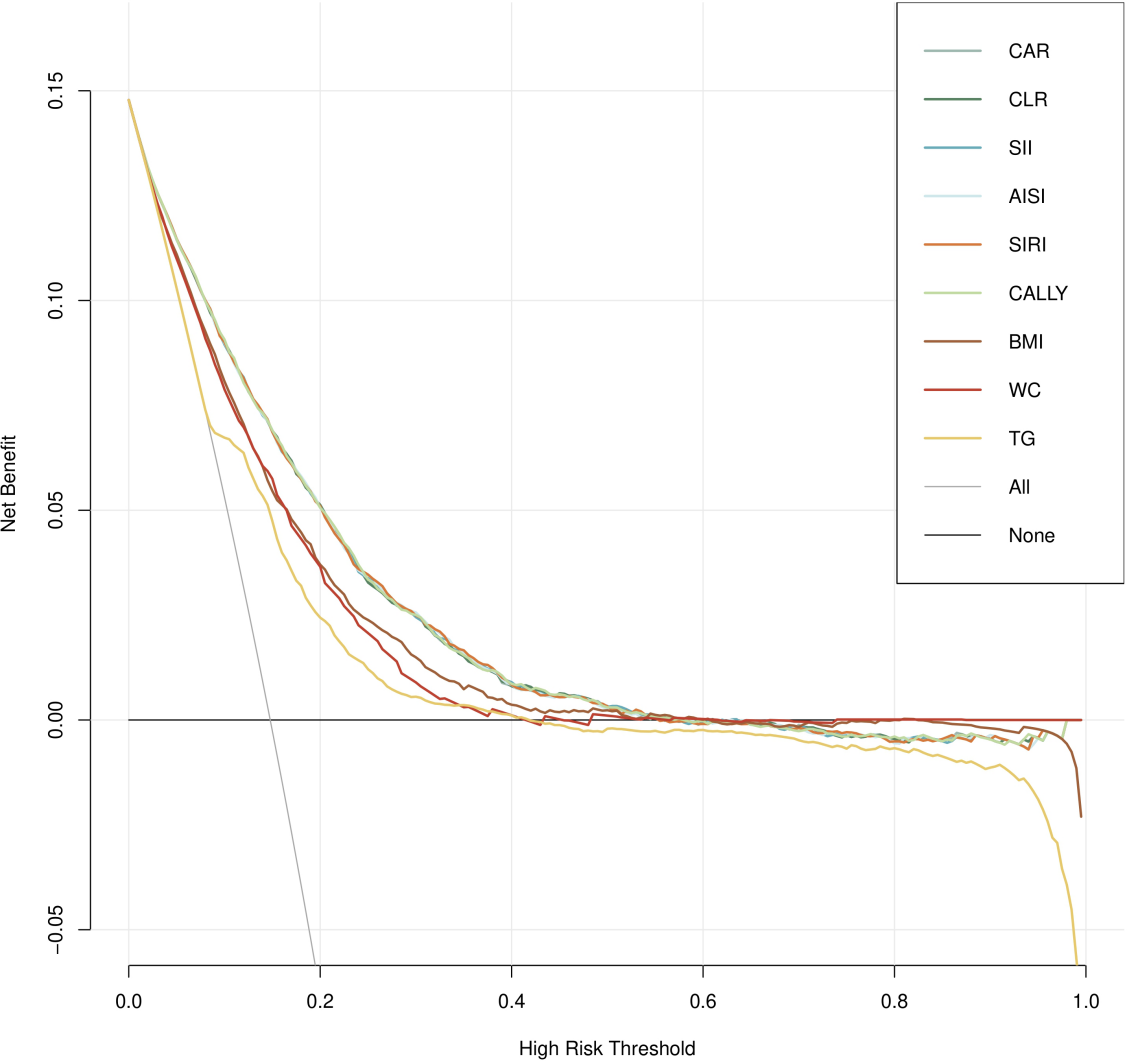
Decision curve analysis (DCA) for composite systemic inflammation indicators (CAR, CLR, SII, AISI, SIRI and CALLY and MAFLD risk in cohort study.

**Figure 4** illustrated the decision curves for these indicators to predict MAFLD in cohort study. It can be seen that, compared with traditional indicators (BMI, WC, TG), inflammation-related indicators, after taking into account gender, age, hypertension and diabetes, BMI, WC, FPG and blood lipids can achieve a more effective balance between intervention and risk at specific threshold probabilities, providing the greatest benefit for clinical decision-making.

### Subgroup analysis

**Figure 5:**
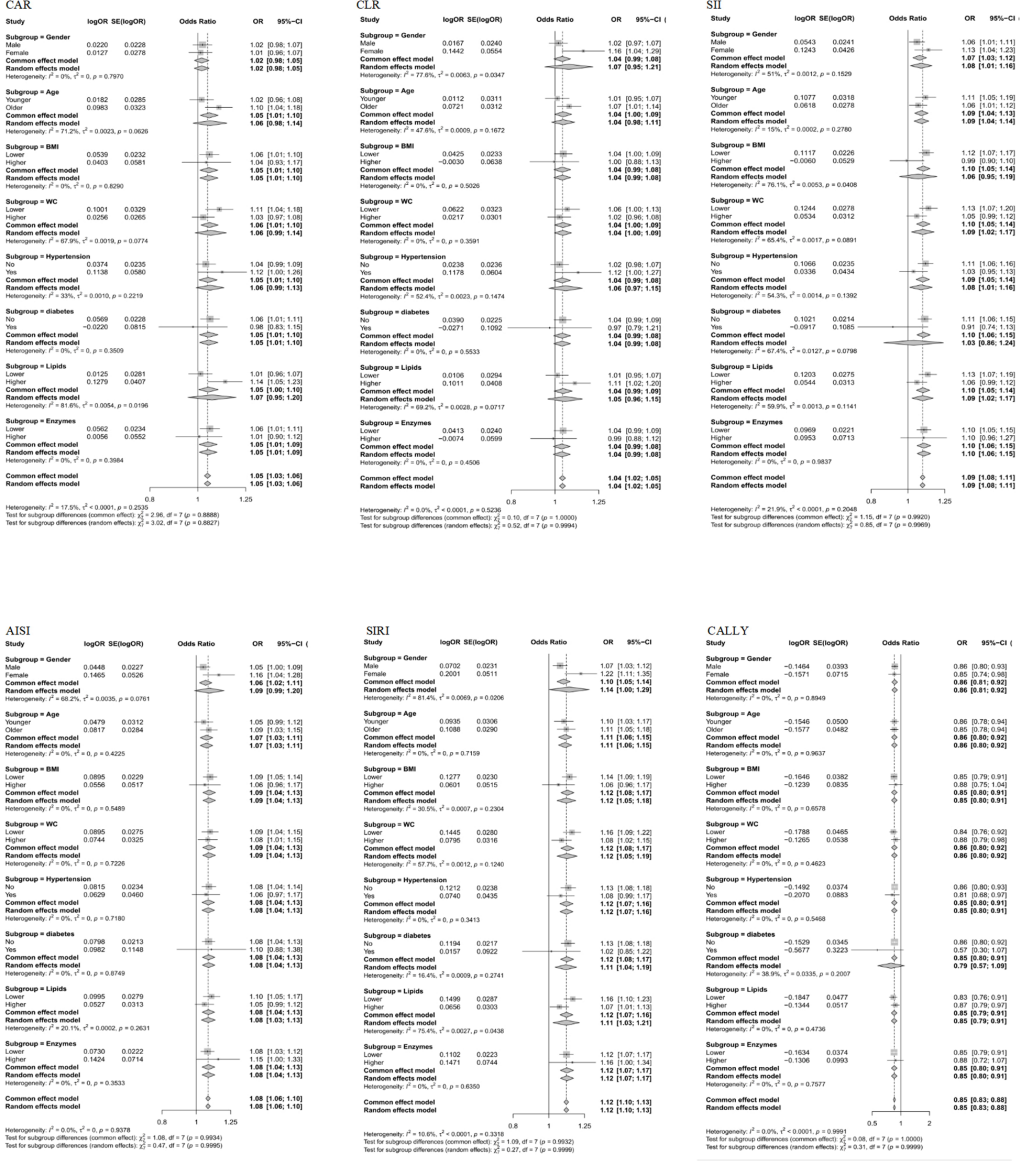
Subgroup analyses of the associations between composite systemic inflammation indicators (CAR, CLR, SII, AISI, SIRI and CALLY) and MAFLD risk in case-control study. CAR: CRP-to-albumin ratio;CLR: CRP-to-lymphocyte ratio; SII: Systemic immune-inflammation index; AISI: aggregate index of systemic inflammation; SIRI: systemic inflammation response index; CALLY: Controlling nutritional status and lymphocyte index; BMI: body mass index; WC: waist circumference; CRP: C-reactive protein. ORs (95%CI) were multi-adjusted for the same variables as model 2 in **Table 2**.

**Figure 6:**
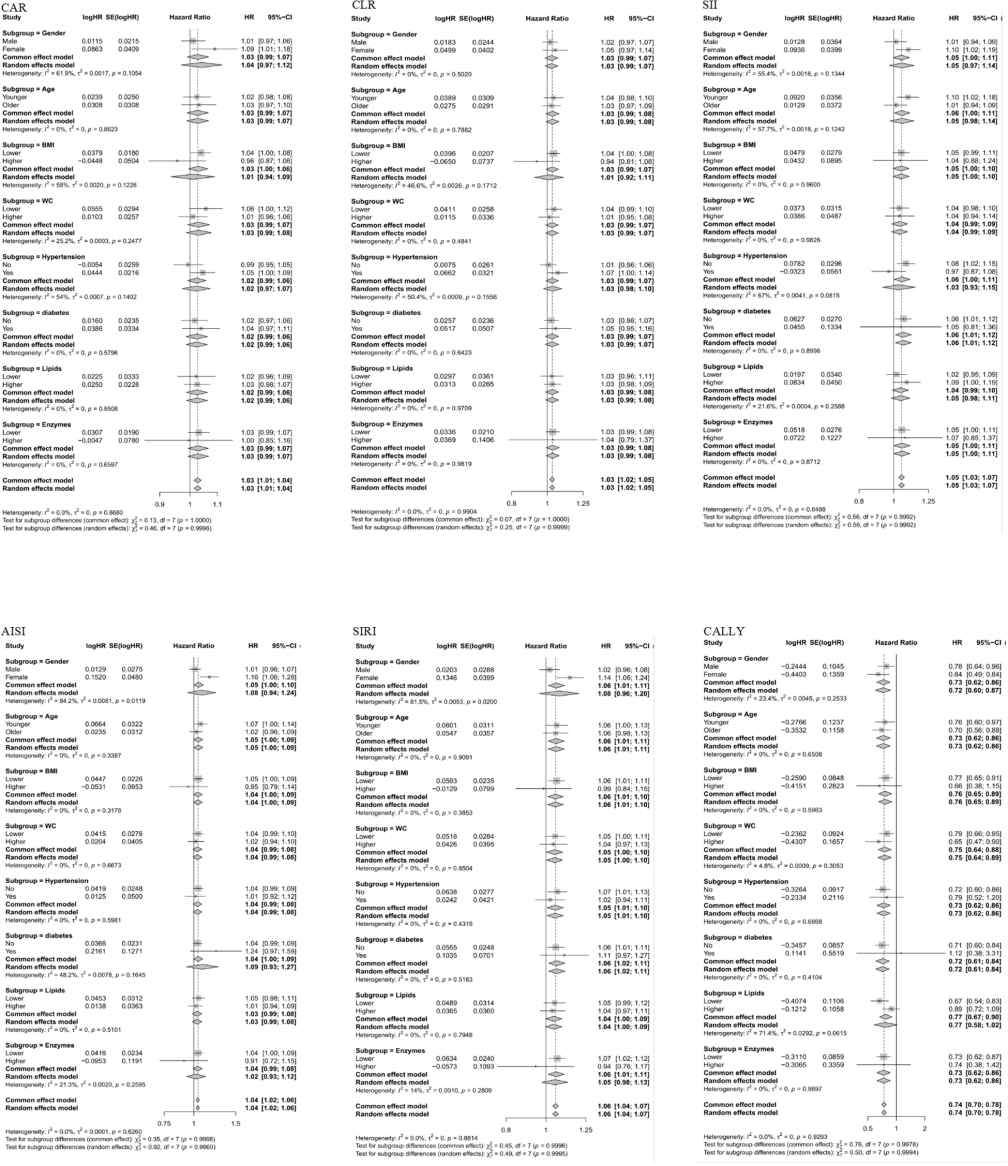
Subgroup analyses of the associations between composite systemic inflammation indicators (CAR, CLR, SII, AISI, SIRI and CALLY) and MAFLD risk in case-control study. CAR: CRP-to-albumin ratio;CLR: CRP-to-lymphocyte ratio; SII: Systemic immune-inflammation index; AISI: aggregate index of systemic inflammation; SIRI: systemic inflammation response index; CALLY: Controlling nutritional status and lymphocyte index; BMI: body mass index; WC: waist circumference; CRP: C-reactive protein. HRs (95%CI) were multi-adjusted for the same variables as model 2 in **Table 3**.

The subgroup analysis of the relationship between six composite immune inflammation indices and MAFLD in case-control and cohort studies is shown in **Figures 5**, **6**. It is worth noting that only SIRI was at risk for women (OR = 1.22, 95% CI: 1.11-1.35; HR = 1.14, 95%: 1.06-1.24) in both case-control and cohort studies was higher than that of men (OR = 1.07, 95% CI: 1.03-1.12; HR = 1.02, CI: 0.96-1.08), and it was heterogeneous (*P* < 0.05). In addition, a case-control study found that there were inter-group differences in the association between the composite immune inflammation index and MAFLD in the lipid subgroup (CAR and SIRI), gender subgroup (CLR), and BMI subgroup (SII) (*P* < 0.05). Cohort studies have shown that there are inter-group differences in the association between AISI and MAFLD in gender subgroups (*P* < 0.05).

## Discussion

This study analyzed peripheral blood inflammation-related indicators (including lymphocytes, neutrophils, platelets, immunoglobulins, etc.) to confirm that there is a complex inflammatory state involving thrombosis, innate immunity and adaptive immune activation in MAFLD patients. The findings of this study closely link MAFLD to the systemic inflammatory state, not only reflecting the local inflammatory response of the liver, but also revealing that MAFLD is a systemic low-grade chronic inflammatory disease. This systemic inflammatory state is very likely to be a key pathological bridge connecting MAFLD with other complications, such as cardiovascular diseases and type 2 diabetes. However, the origin of this inflammatory state is far from being singular. The core driving force is Lipotoxicity caused by metabolic disorders. Excessive accumulation of liver lipids, especially Free fatty acids (FFAs), Free Cholesterol and Ceramides, not only leads to endoplasmic reticulum stress (ERS) and mitochondrial dysfunction, but also generates a large amount of reactive oxygen species (ROS) (23). It can more directly act as a damage-associated molecular model (DAMPs) to activate the natural immune sentinels in the liver - Kupffer Cells. Activated Kupffer cells recruit neutrophils and monocytes in circulation by releasing a large number of pro-inflammatory factors such as TNF-α, IL-1β, and IL-6 (9, 12). Activated hepatic stellate cells (HSCs) are not only the main effector cells of fibrosis, but also can secrete inflammatory mediators themselves. What is more notable is that the adaptive immune system is deeply involved. The infiltration and activation of CD4+ and CD8+ T cells, and even B cells in the liver indicate that the inflammation related to MAFLD has antigen-specific characteristics (6, 11).

This study also verified the significance of gender and age as inflammatory regulatory factors. Due to the protective effect of estrogen, the number of T cells and T-regulatory cells in the body increases, and the development process of liver inflammation is delayed, resulting in a significantly lower incidence of NAFLD in premenopausal women than in men of the same age. Animal studies conducted by Ganz, M et al. also found that a high-fat diet only causes steatohepatitis and inflammasome activation in male mice (24). Subgroup analysis in this study also showed that SIRI, as a composite inflammation index, poses a higher risk for women than men in case-control and cohort studies. Considering that the average age of the study population was 45, the protective effect of estrogen may be one of the reasons why women have a higher risk than men. This result is also consistent with the results obtained from the NHANES population in 2024 (25).The “Inflammaging” brought about by aging - that is, the persistent low activation state of the innate immune system related to age - works in synergy with metabolic stress, significantly exacerbating liver inflammatory damage and oxidative stress levels in elderly patients, which explains the higher risk of disease progression (26–28). Relevant studies have also shown that the subjects in the longevity group exhibited lower lipid peroxidation inflammation, a fact that also indicates the importance of anti-inflammation in anti-aging (29).

Hypertension has now been proven to be a low-grade vascular inflammatory disease (30). The pro-inflammatory factors (such as TNF-α, IL-1β, IL-6) released by activated Kupffer cells and infiltrating immune cells in the liver of MAFLD enter the circulation, which can systematically activate vascular endothelial cells, promote the expression of adhesion molecules (such as VCAM-1, ICAM-1), and recruit monocytes to infiltrate the vascular wall. It can cause vascular inflammation, oxidative stress and endothelial dysfunction, thereby increasing peripheral vascular resistance and driving up blood pressure. There is a close pathophysiological connection between obesity and the pathogenesis of MAFLD (2, 10, 31). Its essence is a vicious cycle of systemic metabolic inflammation and intrahepatic lipotoxicity caused by adipose tissue dysfunction. When the adipose tissue of the liver (especially visceral fat) exceeds its storage capacity, hypertrophic adipocytes enter a hypoxic state and endoplasmic reticulum stress, and the secretion of pro-inflammatory adipokines (such as TNF-α, IL-6, MCP-1) increases. Dysfunctional adipose tissue recruits a large number of macrophages, further amplifying the inflammatory response. Meanwhile, the enhanced lipolysis caused by insulin resistance in adipose tissue leads to the continuous transportation of excessive free fatty acids (FFAs) to the liver, exceeding the β -oxidation capacity of liver cells and resulting in lipid deposition within liver cells. These accumulated lipids (especially free cholesterol, ceramides, etc.) are lipid-toxic, which can induce mitochondrial dysfunction, oxidative stress and endoplasmic reticulum stress, further activate inflammatory signaling pathways and promote liver cell damage. All of this explains why weight management and prevention of the three highs are effective means of treating MAFLD.

This study found that using composite immune-inflammatory-related indicators alone as biomarkers is not ideal (AUC ranging from 0.5 to 0.7), which is consistent with findings from the NHANES study (25). However, when considering gender, age, and metabolism-related indicators, the diagnostic capability significantly improved (AUC over 0.5, sensitivity >75%, specificity >65%). This also indirectly suggests that MAFLD is not merely a concurrent phenomenon of metabolic diseases such as obesity and diabetes, but it also implies that if only the patient’s blood glucose, blood lipids, and blood pressure are controlled without anti-inflammatory treatment, MAFLD itself can continue to promote systemic chronic inflammation. DCA analysis also suggests that under the same conditions, compared to controlling obesity and blood lipids alone, simultaneously regulating inflammation, obesity, blood pressure, blood glucose, and blood lipids can achieve greater benefits. In addition, this result also reveals high-risk groups with “thin MAFLD” or “metabolically healthy MAFLD” (32). Such patients may not be obese or have typical metabolic syndrome, but due to having MAFLD, the level of chronic inflammation in their bodies remains high, and the risk of cardiovascular diseases and liver fibrosis in the future still increases significantly.

This study also found that the relationship between MAFLD and inflammatory markers significantly slowed down after exceeding a certain cut-off value. This also explains why the dose-response effect of inflammation and MAFLD is not very strong. However, this nonlinear feature is highly consistent with the pathophysiological mechanism of MAFLD (11, 33, 34). In the early stage of MAFLD, liver tissue is mainly characterized by simple steatosis and mild inflammatory infiltration, and the level of inflammation in this stage increases rapidly with the accumulation of lipids in the liver and the intensification of insulin resistance. However, this process is largely reversible, and measures such as lifestyle interventions and metabolic improvements can effectively alleviate the inflammatory response and even achieve histological reversal (35). As the disease progresses, especially when liver damage persists and enters the fibrosis stage, a complex network of interactions between inflammation and fibrosis is formed, and the liver microenvironment is fundamentally changed. At this time, although anti-inflammatory treatment is still important, it is difficult to achieve complete reversal of fibrosis by controlling inflammation alone, and the disease has entered a plateau period of relatively stable but continuous progression. This underscores the urgency and window period for early intervention in MAFLD, where interventions may yield greater therapeutic benefits and reversibility before inflammation grows rapidly but does not yet reach the plateau threshold. Secondly, the treatment strategy needs to be adjusted accordingly after the disease enters the plateau, and the focus should be expanded from simple anti-inflammatory to multi-dimensional comprehensive interventions such as anti-fibrosis and complication management.

Although there are many studies on inflammation-related indicators and fatty liver, the following problems are common: (1) the study is still the definition of NAFLD, not MAFLD; (2) studies are limited to a single inflammatory marker; (3) studies rarely perform multiple validation in both case-control and cohort populations; (4) Most of the studies came from databases, and most of the population was non-Asian. This study made up for these shortcomings by exploring the relationship between multiple inflammation-related indicators (CAR, CLR, SII, AISI, SIRI and CALLY) and MAFLD in case-control and cohort studies in China. Of course, this study also has many shortcomings. Firstly, the imaging diagnostic criteria for MAFLD are based on ultrasound. Compared with liver biopsy, this may reduce the detection rate of fatty liver. Previous studies have shown that traditional ultrasound has a better diagnostic effect on steatosis of ≥30% (36).Although we have added FLI as a quantitative diagnostic reference for steatosis to make up for this deficiency, more precise diagnostic methods (such as ultrasound attenuation coefficient, controlled attenuation parameters, etc.) are still needed. Second, unlike the active follow-up method adopted in other studies, the follow-up data in this study was passively collected from the health screening cohort, which may lead to bias in follow-up information. However, considering the annual population health check-up cycle, the currently lost follow-up population still has the possibility of returning in the future. Third, single-center data may limit the generalizability of this study. Fourth, this study did not collect data on dietary.

## Conclusion

This large-scale, two-phase study provides strong evidence for the relationship between systemic immune inflammatory activation and MAFLD in the Chinese population. These nonlinear associations persist, especially after adjusting for gender, age, and metabolism-related confounders, suggesting that the inflammatory state in MAFLD is not just a collateral phenomenon of obesity or dyslipidemia, but may represent an independent pathological pathway. The nonlinear threshold-effect relationship suggests a complex pathophysiology, reinforcing the concept of MAFLD as a systemic metabolic inflammatory disease and highlighting the potential of anti-inflammatory strategies, especially in the early, reversible stages of the disease before the inflammatory response arrests.

## Data Availability

The raw data supporting the conclusions of this article will be made available by the authors, without undue reservation.
