# Supplementary material for: Associations between composite systemic inflammation indicators(CAR, CLR, SII, AISI, SIRI, and CALLY) and metabolic dysfunction-associated fatty liver disease (MAFLD): evidence from a two-stage study in China
Source: Front Immunol. 2025 Nov 21;16:1702567. doi: 10.3389/fimmu.2025.1702567 (PMC12678147; doi:10.3389/fimmu.2025.1702567)
Supplement: Supplementary file 1 [file DataSheet1.docx]

**Supplymentary Table 1. Baseline characteristics between participants lost to follow-up and those who completed follow-up in cohort study**

|  | **Overall** | **Complete** | **Lost to follow-up** | P value |
| --- | --- | --- | --- | --- |
| **N** | 18861 | 8627 | 10234 |  |
| **CAR** | 0.21 (0.12,0.38) | 0.20 (0.13, 0.34) | 0.22 (0.11,0.42) | 0.38 |
| **CLR** | 0.43 (0.24,0.81) | 0.42 (0.27, 0.72) | 0.45 (0.21,0.89) | 0.14 |
| **SII** | 357.31 (266.94,478.44) | 357.35 (267.38, 471.91) | 357.32 (266.49,484.56) | 0.23 |
| **AISI** | 0.64 (0.46,0.89) | 0.64 (0.46, 0.88) | 0.64 (0.46,0.90) | 0.21 |
| **SIRI** | 147.44 (100.01,219.90) | 147.44 (101.71, 217.82) | 147.45 (98.59,221.30) | 0.54 |
| **CALLY** | 10.33 (6.26,16.55) | 10.51 (6.14, 16.50) | 10.12 (6.32,16.57) | 0.14 |
| **Gender** |  |  |  | 0.66 |
| **Male, n (%)** | 8086 (42.87%) | 3714 (43.06%) | 4372 (42.72%) |  |
| **Female, n (%)** | 10775 (57.13%) | 4913 (56.94%) | 5862 (57.28%) |  |
| **Age, years** | 40.11 (32.01,51.11) | 39.79 (32.05, 50.70) | 40.51 (31.97,51.39) | 0.59 |
| **BMI, kg/m2** | 22.70 (20.80,24.70) | 22.70 (20.70, 24.60) | 22.80 (20.80,24.70) | 0.13 |
| **WC, cm** | 79.00 (72.00,85.00) | 79.00 (72.00, 85.00) | 79.00 (72.00,85.00) | 0.13 |
| **AST, U/L** | 19.90 (16.90,23.90) | 20.00 (17.00, 23.80) | 19.90 (16.90,23.90) | 0.10 |
| **ALT, U/L** | 15.60 (11.60,22.40) | 15.70 (11.50, 22.10) | 15.60 (11.60,22.60) | 0.13 |
| **TC, mmol/L** | 4.90 (4.33,5.56) | 4.89 (4.33, 5.52) | 4.91 (4.32,5.59) | 0.22 |
| **TG, mmol/L** | 1.06 (0.78,1.50) | 1.06 (0.78, 1.49) | 1.07 (0.78,1.51) | 0.42 |
| **HDL-C, mmol/L** | 1.38 (1.18,1.62) | 1.38 (1.19, 1.61) | 1.38 (1.18,1.62) | 0.72 |
| **LDL-C, mmol/L** | 2.88 (2.43,3.36) | 2.87 (2.44, 3.35) | 2.88 (2.43,3.37) | 0.64 |
| **Hypertension** |  |  |  | 0.41 |
| **Yes, n (%)** | 2248 (11.92%) | 1047 (12.14%) | 1201 (11.74%) |  |
| **No, n (%)** | 16613 (88.08%) | 7580 (87.86%) | 9033 (88.26%) |  |
| **Diabetes** |  |  |  | 0.46 |
| **Yes, n (%)** | 377 (2.00%) | 180 (2.09%) | 197 (1.92%) |  |
| **No, n (%)** | 18484 (98.00%) | 8447 (97.91%) | 10037 (98.08%) |  |
| **CRP, mg/L** | 0.92 (0.54,1.71) | 0.89 (0.59, 1.51) | 0.96 (0.49,1.89) | 0.18 |
| **Neutrophil count, 10⁹ cells/L** | 3.22 (2.62,3.96) | 3.23 (2.64, 3.95) | 3.20 (2.59,3.97) | 0.09 |
| **Lymphocyte count, 10⁹ cells/L** | 2.12 (1.76,2.54) | 2.12 (1.76, 2.55) | 2.12 (1.76,2.53) | 0.32 |
| **Platelet count, 10⁹ cells/L** | 234.00 (200.00,273.00) | 234.00 (202.00, 273.00) | 234.00 (199.00,273.00) | 0.30 |
| **Monocyte count, 10⁹ cells/L** | 0.41 (0.34,0.51) | 0.42 (0.34, 0.51) | 0.41 (0.33,0.51) | 0.58 |

Note:

Numerical variables are presented as median (lower quartile, upper quartile), and categorical variables are presented as count (percentage).

CAR: CRP-to-albumin ratio; CLR: CRP-to-lymphocyte ratio; SII: Systemic immune-inflammation index; AISI: aggregate index of systemic inflammation; SIRI: systemic inflammation response index; CALLY: Controlling nutritional status and lymphocyte index; BMI: body mass index; WC: waist circumference; AST: aspartate aminotransferase; ALT: alanine aminotransferase; TG: triglycerides; TC: total cholesterol; HDL-C: high density lipoprotein-cholesterol; LDL-C: low density lipoprotein-cholesterol; CRP: C-reactive protein.

**Supplymentary Table 2. Sensitivity analysis of the case-control study: further adjusted for ALT and AST**

| **Case-control** | **Cases/Controls** | **Model 3** | ***P* value** | **Model 4** | ***P* value** |
| --- | --- | --- | --- | --- | --- |
| CAR |  |  |  |  |  |
| CAR per-sd increase | 7894/7894 | 1.05 (1.01,1.09) | **0.02** | 1.06 (1.01,1.10) | **0.01** |
| Q1 | 1137/2807 | Reference | Reference | Reference | Reference |
| Q2 | 1842/2110 | 1.37 (1.22,1.54) | **<0.01** | 1.40 (1.26,1.57) | **<0.01** |
| Q3 | 2230/1715 | 1.59 (1.41,1.79) | **<0.01** | 1.69 (1.51,1.89) | **<0.01** |
| Q4 | 2685/1262 | 1.83 (1.61,2.07) | **<0.01** | 1.94 (1.72,2.19) | **<0.01** |
| *P* for trend |  |  | **<0.01** |  | **<0.01** |
| CLR |  |  |  |  |  |
| CLR per-sd increase | 7894/7894 | 1.04 (0.99,1.08) | 0.10 | 1.04 (1.00,1.08) | 0.08 |
| Q1 | 1317/2632 | Reference | Reference | Reference | Reference |
| Q2 | 1817/2128 | 1.16 (1.03,1.30) | **0.01** | 1.21 (1.08,1.35) | **<0.01** |
| Q3 | 2180/1766 | 1.30 (1.15,1.46) | **<0.01** | 1.37 (1.22,1.53) | **<0.01** |
| Q4 | 2580/1368 | 1.60 (1.41,1.80) | **<0.01** | 1.69 (1.50,1.90) | **<0.01** |
| *P* for trend |  |  | **<0.01** |  | **<0.01** |
| SII |  |  |  |  |  |
| SII per-sd increase | 7894/7894 | 1.10 (1.05,1.14) | **<0.01** | 1.07 (1.03,1.12) | **<0.01** |
| Q1 | 1746/2201 | Reference | Reference | Reference | Reference |
| Q2 | 1865/2082 | 0.99 (0.89,1.11) | 0.91 | 0.98 (0.87,1.09) | 0.69 |
| Q3 | 2111/1836 | 1.22 (1.09,1.37) | **<0.01** | 1.18 (1.05,1.32) | **<0.01** |
| Q4 | 2172/1775 | 1.28 (1.14,1.44) | **<0.01** | 1.20 (1.07,1.34) | **<0.01** |
| *P* for trend |  |  | **<0.01** |  | **<0.01** |
| AISI |  |  |  |  |  |
| AISI per-sd increase | 7894/7894 | 1.08 (1.04,1.13) | **<0.01** | 1.09 (1.04,1.13) | **<0.01** |
| Q1 | 1652/2374 | Reference | Reference | Reference | Reference |
| Q2 | 1972/2053 | 1.12 (1.00,1.26) | **0.04** | 1.13 (1.01,1.26) | **0.03** |
| Q3 | 2162/1862 | 1.21 (1.08,1.36) | **<0.01** | 1.22 (1.09,1.36) | **<0.01** |
| Q4 | 2265/1761 | 1.25 (1.11,1.40) | **<0.01** | 1.27 (1.13,1.42) | **<0.01** |
| *P* for trend |  |  | **<0.01** |  | **<0.01** |
| SIRI |  |  |  |  |  |
| SIRI per-sd r increase | 7894/7894 | 1.12 (1.07,1.17) | **<0.01** | 1.12 (1.07,1.16) | **<0.01** |
| Q1 | 1548/2399 | Reference | Reference | Reference | Reference |
| Q2 | 1889/2058 | 1.18 (1.05,1.32) | **<0.01** | 1.16 (1.04,1.30) | **<0.01** |
| Q3 | 2131/1816 | 1.32 (1.18,1.48) | **<0.01** | 1.31 (1.17,1.47) | **<0.01** |
| Q4 | 2326/1621 | 1.46 (1.30,1.64) | **<0.01** | 1.45 (1.29,1.62) | **<0.01** |
| *P* for trend |  |  | **<0.01** |  | **<0.01** |
| CALLY |  |  |  |  |  |
| CALLY per-sd increase | 7894/7894 | 0.85 (0.80,0.91) | **<0.01** | 0.84 (0.78,0.89) | **<0.01** |
| Q1 | 2559/1387 | Reference | Reference | Reference | Reference |
| Q2 | 2172/1774 | 0.84 (0.75,0.94) | **<0.01** | 0.84 (0.75,0.94) | **<0.01** |
| Q3 | 1834/2112 | 0.76 (0.68,0.85) | **<0.01** | 0.76 (0.68,0.85) | **<0.01** |
| Q4 | 1328/2618 | 0.65 (0.58,0.74) | **<0.01** | 0.63 (0.56,0.70) | **<0.01** |
| *P* for trend |  |  | **<0.01** |  | **<0.01** |

Note:

Data are presented as count, OR (95%CI).

Model 3 was adjusted for gender and age, hypertension, diabetes, BMI, WC, TC, TG, HDL-C, LDL-C, FPG, ALT, AST; Model 3 was adjusted for gender and age, hypertension, diabetes, BMI, WC, TC, TG, HDL-C, LDL-C, FPG, smoking and exercise.

CAR: CRP-to-albumin ratio; CLR: CRP-to-lymphocyte ratio; SII: Systemic immune-inflammation index; AISI: aggregate index of systemic inflammation; SIRI: systemic inflammation response index; CALLY: Controlling nutritional status and lymphocyte index; BMI: body mass index; WC: waist circumference; TG: triglycerides; TC: total cholesterol; HDL-C: high density lipoprotein-cholesterol; LDL-C: low density lipoprotein-cholesterol; CRP: C-reactive protein; AST: aspartate aminotransferase; ALT: alanine aminotransferase.

**Supplymentary Table 3. Sensitivity analysis of the case-control study: further adjusted for Smoking and Physical Exercise**

| **Cohort** | **N/Cases** | **Person-years** | **Model3** | ***P* value** | **Model4** | ***P* value** |
| --- | --- | --- | --- | --- | --- | --- |
| CAR |  |  |  |  |  |  |
| CAR per-sd increase | 8627/1275 | 21352 | 1.02 (0.99,1.06) | 0.23 | 1.02 (0.99,1.06) | 0.22 |
| Q1 | 2157/138 | 5532 | Reference | Reference | Reference | Reference |
| Q2 | 2166/278 | 5472 | 1.40 (1.14,1.72) | **<0.01** | 1.43 (1.16,1.75) | **<0.01** |
| Q3 | 2147/371 | 5284 | 1.49 (1.22,1.83) | **<0.01** | 1.52 (1.25,1.86) | **<0.01** |
| Q4 | 2157/488 | 5065 | 1.80 (1.48,2.20) | **<0.01** | 1.83 (1.50,2.23) | **<0.01** |
| *P* for trend |  |  |  | **<0.01** |  | **<0.01** |
| CLR |  |  |  |  |  |  |
| CLR per-sd increase | 8627/1275 | 21352 | 1.03 (0.99,1.07) | 0.17 | 1.03 (0.99,1.07) | 0.18 |
| Q1 | 170/2161 | 5520 | Reference | Reference | Reference | Reference |
| Q2 | 284/2153 | 5458 | 1.21 (0.99,1.46) | 0.06 | 1.23 (1.02,1.49) | **0.03** |
| Q3 | 372/2156 | 5266 | 1.50 (1.25,1.81) | **<0.01** | 1.55 (1.28,1.86) | **<0.01** |
| Q4 | 449/2157 | 5108 | 1.53 (1.27,1.84) | **<0.01** | 1.56 (1.29,1.87) | **<0.01** |
| *P* for trend |  |  |  | **<0.01** |  | **<0.01** |
| SII |  |  |  |  |  |  |
| SII per-sd increase | 8627/1275 | 21352 | 1.06 (1.01,1.12) | **0.02** | 1.06 (1.00,1.11) | **0.03** |
| Q1 | 245/2157 | 5252 | Reference | Reference | Reference | Reference |
| Q2 | 324/2157 | 5304 | 1.23 (1.04,1.46) | **0.01** | 1.26 (1.07,1.49) | **<0.01** |
| Q3 | 337/2156 | 5292 | 1.39 (1.18,1.64) | **<0.01** | 1.37 (1.16,1.62) | **<0.01** |
| Q4 | 369/2157 | 5504 | 1.26 (1.07,1.49) | **<0.01** | 1.26 (1.07,1.49) | **<0.01** |
| *P* for trend |  |  |  | **<0.01** |  | **<0.01** |
| AISI |  |  |  |  |  |  |
| AISI per-sd increase | 8627/1275 | 21352 | 1.04 (1.00,1.09) | 0.07 | 1.04 (1.00,1.09) | 0.05 |
| Q1 | 200/2157 | 5294 | Reference | Reference | Reference | Reference |
| Q2 | 299/2157 | 5292 | 1.26 (1.05,1.51) | **0.01** | 1.25 (1.04,1.50) | **0.01** |
| Q3 | 371/2156 | 5324 | 1.38 (1.16,1.64) | **<0.01** | 1.36 (1.14,1.62) | **<0.01** |
| Q4 | 405/2157 | 5442 | 1.41 (1.18,1.67) | **<0.01** | 1.41 (1.19,1.68) | **<0.01** |
| *P* for trend |  |  |  | **<0.01** |  | **<0.01** |
| SIRI |  |  |  |  |  |  |
| SIRI per-sd r increase | 8627/1275 | 21352 | 1.06 (1.01,1.11) | **0.01** | 1.06 (1.01,1.11) | **0.01** |
| Q1 | 203/2157 | 5290 | Reference | Reference | Reference | Reference |
| Q2 | 305/2157 | 5304 | 1.39 (1.16,1.67) | **<0.01** | 1.37 (1.14,1.64) | **<0.01** |
| Q3 | 369/2156 | 5366 | 1.40 (1.18,1.67) | **<0.01** | 1.40 (1.18,1.66) | **<0.01** |
| Q4 | 598/2157 | 5392 | 1.48 (1.25,1.76) | **<0.01** | 1.46 (1.23,1.74) | **<0.01** |
| *P* for trend |  |  |  | **<0.01** |  | **<0.01** |
| CALLY |  |  |  |  |  |  |
| CALLY per-sd increase | 8627/1275 | 21352 | 0.72 (0.61,0.85) | **<0.01** | 0.71 (0.60,0.84) | **<0.01** |
| Q1 | 451/2157 | 5134 | Reference | Reference | Reference | Reference |
| Q2 | 370/2157 | 5264 | 0.94 (0.82,1.08) | 0.38 | 0.95 (0.83,1.10) | 0.50 |
| Q3 | 281/2156 | 5442 | 0.80 (0.69,0.94) | **<0.01** | 0.81 (0.69,0.94) | **<0.01** |
| Q4 | 173/2157 | 5513 | 0.66 (0.55,0.79) | **<0.01** | 0.65 (0.54,0.78) | **<0.01** |
| *P* for trend |  |  |  | **<0.01** |  | **<0.01** |

Note:

Data are presented as count, HR (95%CI).

Model 3 was adjusted for gender and age, hypertension, diabetes, BMI, WC, TC, TG, HDL-C, LDL-C, FPG, ALT, AST; Model 3 was adjusted for gender and age, hypertension, diabetes, BMI, WC, TC, TG, HDL-C, LDL-C, FPG, smoking and exercise.

CAR: CRP-to-albumin ratio; CLR: CRP-to-lymphocyte ratio; SII: Systemic immune-inflammation index; AISI: aggregate index of systemic inflammation; SIRI: systemic inflammation response index; CALLY: Controlling nutritional status and lymphocyte index; BMI: body mass index; WC: waist circumference; TG: triglycerides; TC: total cholesterol; HDL-C: high density lipoprotein-cholesterol; LDL-C: low density lipoprotein-cholesterol; CRP: C-reactive protein; AST: aspartate aminotransferase; ALT: alanine aminotransferase.
